# Longterm Followup of a Pediatric Patient with Congenital Abdominal Aortic Aneurysm with Coarctation

**DOI:** 10.3400/avd.cr.22-00067

**Published:** 2022-12-25

**Authors:** Masahide Chikada, Kiyoshi Chiba, Kan Nawata, Masahiro Tomita, Ryuji Nakamura, Satoshi Kinebuchi, Shota Kita, Masahide Komagamine, Misa Kogo, Hiroshi Nishimaki, Takeshi Miyairi

**Affiliations:** 1 Department of Cardiovascular Surgery, St. Marianna University School of Medicine, Kawasaki, Kanagawa, Japan

**Keywords:** congenital, aortic aneurysm, coarctation

## Abstract

Congenital abdominal aortic aneurysm (AAA) with coarctation has been considered an extremely rare condition. In this study, we present a 3-year-old boy, who was diagnosed by chance with congenital AAA at first operation. We replaced the AAA+coarctation with a 6-mm polytetrafluoroethylene (PTFE) graft. Histological examination of the aortic wall revealed no particular abnormalities. Collateral vessels were noted to develop over 14 years of followup. Good blood flow to both lower limbs and no intermittent claudication were observed. After growth, at the age 17, he underwent extra-anatomical bypass using a 12-mm PTFE graft. This is the first report of successful treatment of congenital AAA+coarctation with longterm followup.

## Introduction

Pediatric abdominal aortic aneurysm (AAA) is uncommon; it is known to be usually caused by infection, aortitis, or iatrogenic trauma. Congenital AAA with coarctation is an extremely rare condition that is thought to carry a high mortality rate. In this study, we present a patient with congenital AAA with coarctation, whom we followed up for 14 years.

## Case Report

**Figure 1:**
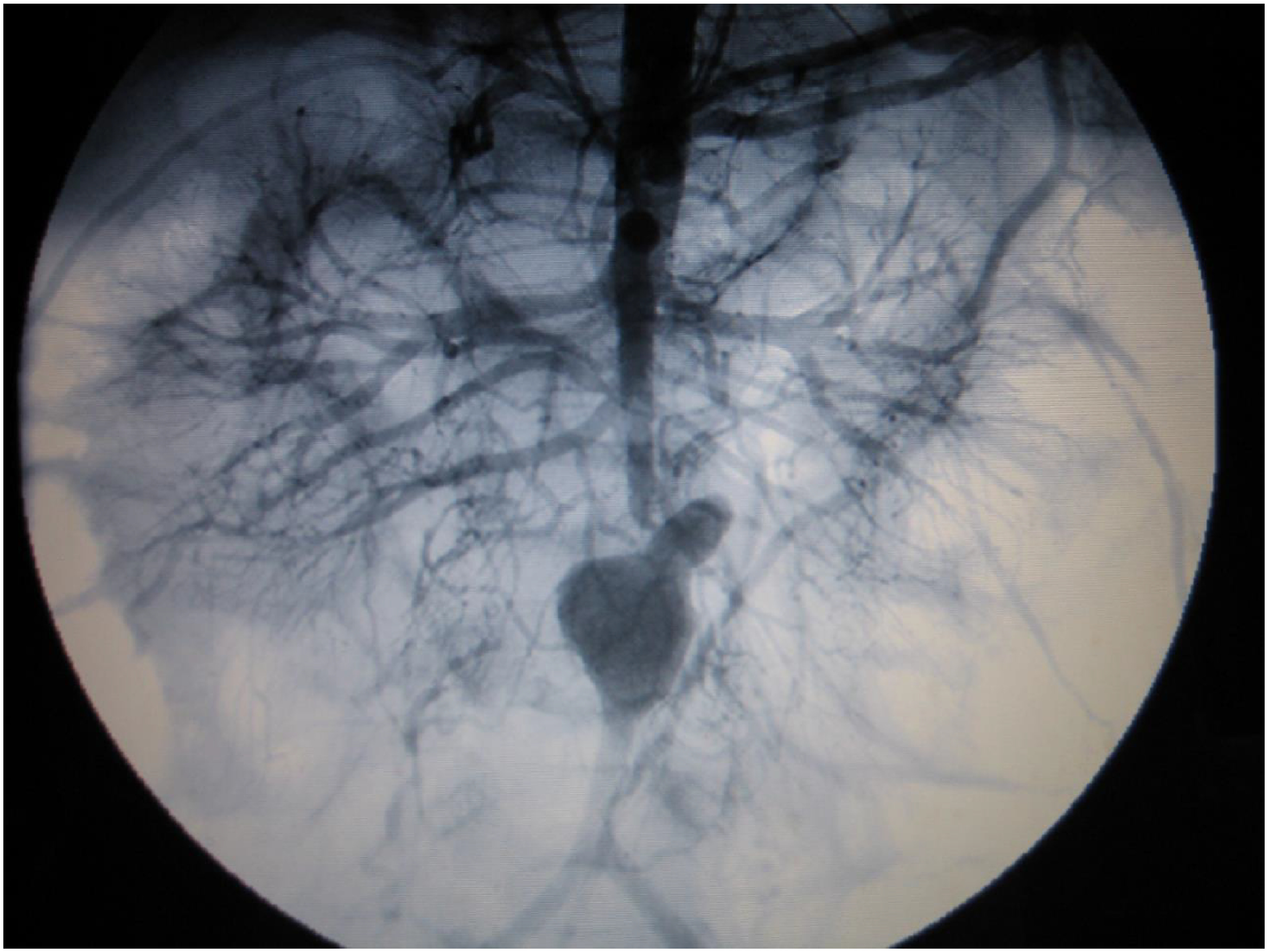
Fig. 1 Abdominal aortic angiography before the first operation. Angiography of the abdominal aorta showed an irregularly shaped abdominal aortic aneurysm (AAA) of 25 mm in size and atypical coarctation of the proximal segment of the AAA.

The male patient was 3 years old during his first operation. He was admitted into our hospital because of a respiratory infection. A physical examination revealed a vascular murmur in his abdomen. Laboratory tests showed no abnormal data, and other general examinations revealed no abnormal findings. He had no history of trauma and no family history of AAA. However, after conducting an enhanced computed tomography (CT) on his abdomen, an irregularly shaped AAA was detected. Angiography of the abdominal aorta was then performed that showed an irregularly shaped AAA of 25 mm in size and atypical coarctation of the proximal segment of the AAA (**Fig. 1**). No additional aneurysms or concomitant diseases were found. The lower extremity ischemia was not found because of well-developing collateral arteries. Because of the potential for risk of rupture or sudden occlusion due to the irregular shape and long narrow segment of the coarctation, we performed graft replacement of the AAA.

**Figure 2:**
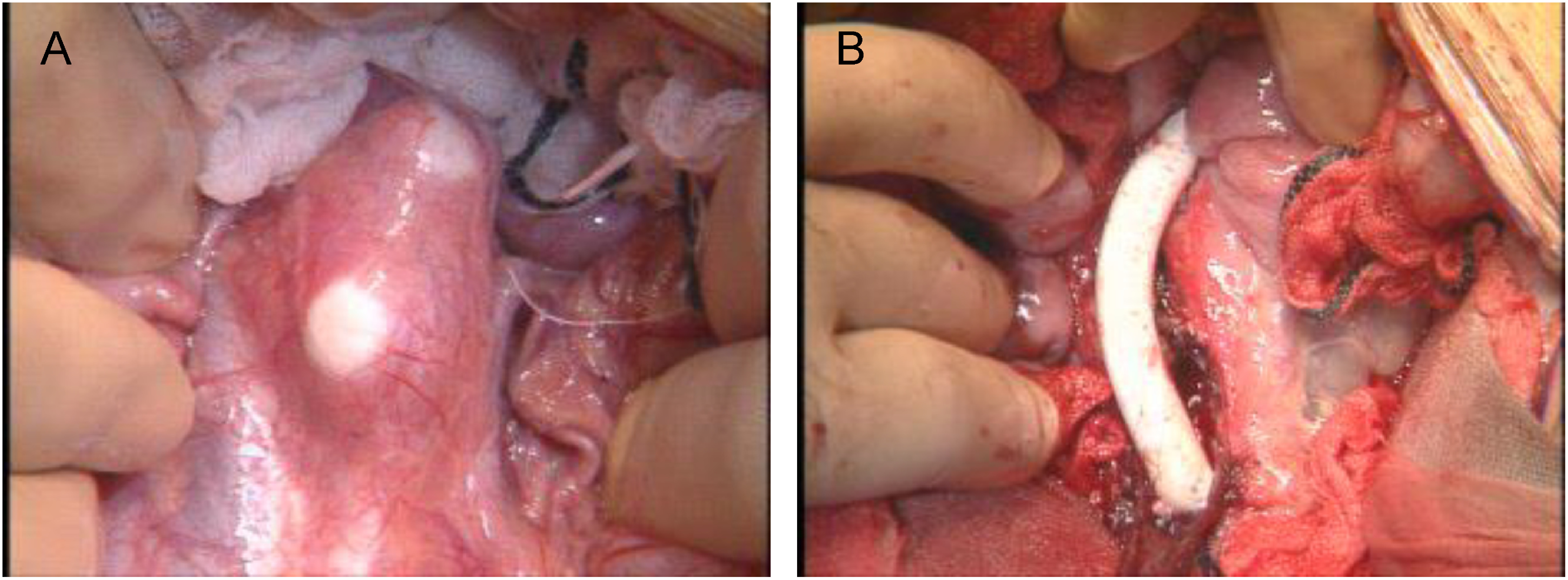
Fig. 2 Operative views of the first operation. (**A**) Operative photo shows an irregularly shaped abdominal aortic aneurysm (AAA) exposed through a midline incision. (**B**) The aortic segment comprising the AAA with coarctation was replaced with a 6-mm polytetrafluoroethylene graft.

During the operation, the AAA was exposed through a midline incision. The operative view showed the irregular shape of the AAA (**Fig. 2A**). We then resected the segment containing both the AAA and coarctation and replaced it with a 6-mm polytetrafluoroethylene (PTFE) graft (**Fig. 2B**). Histological examination of the resected aortic wall revealed no evidence of cystic generation of the media, active aortitis, or infection. The media showed only slight hypertrophic change. The intima showed no calcification, ulceration, thrombosis, or rupture. Special staining was not performed. The AAA was neither a dissection nor a pseudoaneurysm and thus was thought to be a true aneurysm. The boy’s postoperative course was deemed uneventful.

**Figure 3:**
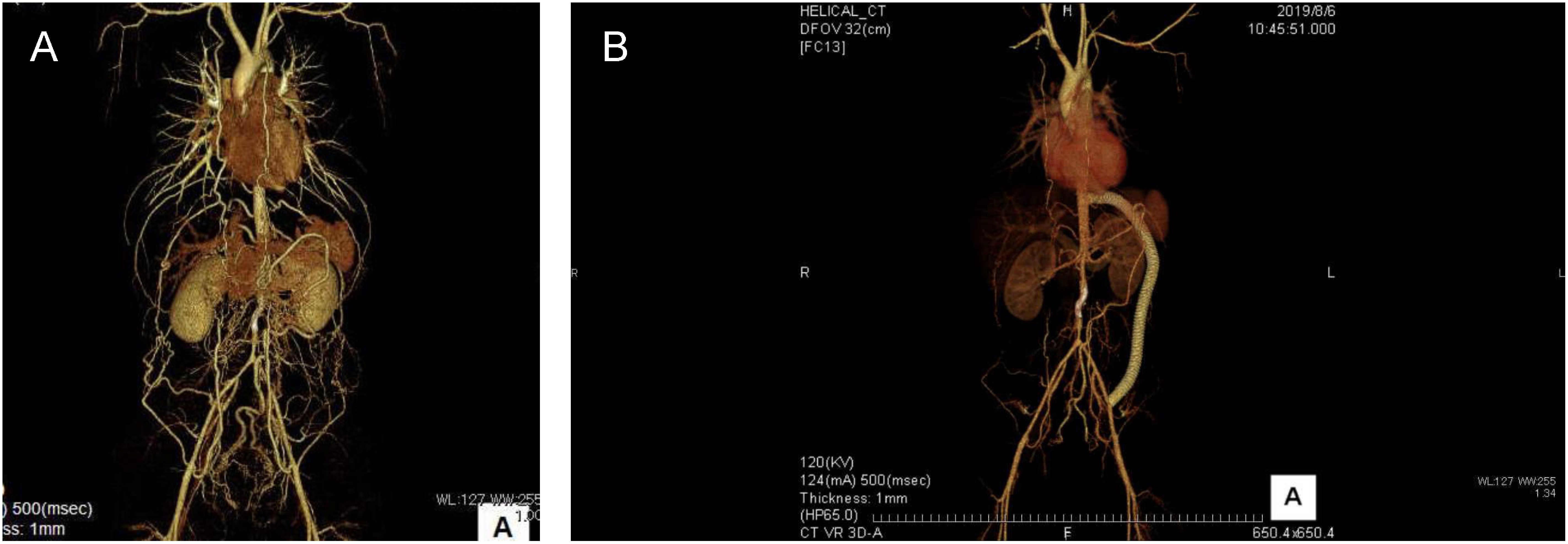
Fig. 3 (**A**) Enhanced computed tomography before the second surgery shows that both the proximal and distal sites of anastomosis are still too narrow, and there are numerous collateral vessels developing. (**B**) Enhanced computed tomography following the second surgery 1 year later. Anterior view shows that both grafts are patent and that numerous collateral vessels have developed.

We did not use drugs to prevent graft occlusion because the patient and his parents refused such drugs. The 6-mm PTFE graft was becoming relatively narrow due to the boy’s growth. He had no pain in his feet during exercise because a number of collateral vessels had developed, and both limbs have grown normally. However, when he stopped growing, he began to feel slight pain in his lower limbs during exercise. Replacement of the graft was thought to be difficult because both the proximal and distal sites of anastomosis were still too narrow (**Fig. 3A**). We therefore decided to perform an extra-anatomical bypass. At the age of 17 year old, he underwent successful implantation of an extra-anatomical bypass with a 12-mm PTFE graft. The pain felt in his lower limbs during exercise disappeared after the second operation. Enhanced CT showed that both grafts were patent, and numerous collateral vessels still existed (**Fig. 3B**). Ankle-brachial indexes were noted to improve (right from 0.68 to 0.91 and left from 0.77 to 0.88) after surgery.

## Discussion

According to one classification system, congenital AAA is defined as a localized truncal form of arterial defect.^1)^ This arterial malformation occurs by developmental arrest of the arterial system in the later embryogenetic stage. The molecular and genetic origins of congenital AAA are almost unknown because of the rarity of congenital AAA. Pediatric AAA is known to be rare; however, its common causes are connective tissue disorders, vasculitis, iatrogenic trauma, and infection. Congenital AAA is idiopathic and has an unknown etiology. One paper^2)^ suggests that congenital AAA occurs from a developmental defect during embryogenesis that causes a narrow segment of the abdominal aorta, which leads to poststenotic turbulent blood flow and subsequent aneurysm formation. Our patient with congenital AAA with coarctation matches this hypothesis because there was a narrow segment of coarctation. Only 32 cases have been reported recently, including our case.^3)^ A review^4)^ showed that males were almost twice as likely to develop congenital AAA than females, half of the patients were neonates and infants, and only 4 of 26 patients were diagnosed at over 3 years of age. Our patient was diagnosed later than normal. One-third of the patients were asymptomatic, and the location of the AAA was infrarenal in 70% of the patients. The mortality rate of congenital AAA rupture and renal failure was as high as around 30%.

A standard approach to the treatment of congenital AAA is yet to be established. Seven of 26 patients underwent conservative treatment because of extensive aneurysms, small vessel diameter, and poor hemodynamic conditions. The mortality of those receiving conservative treatment was noted to be very high: four deaths were reported. Seventeen patients underwent surgical repair, with artificial grafts most frequently chosen for replacement. A Dacron graft was used in six cases, a PTFE graft in three cases, allografts in two cases, and aneurysmorrhaphy in two cases. One case was repaired with native iliac vessels. We had no experience of using a native vessel as a graft, and we were familiar with using a PTFE graft. Thus, we chose a PTFE graft to replace the abdominal aortic aneurysm. The worrisome complications of revascularization were graft stenosis and occlusion. A high risk of occlusion in artificial grafts with a diameter of less than 6 mm is reported.^5)^ The 6-mm PTFE graft used in our case was at the lower allowable selection limit, but an 8-mm graft was too large to anastomose. In the long run, the 6-mm PTFE graft has shown longterm patency. However, a PTFE graft does not grow, and ischemic symptoms in the lower limbs may occur as the patient grows. At the initial surgery, we thought that the second surgery would be the replacement of the graft so we did not choose the extra-anatomical bypass. We have followed our patient regularly in an outpatient clinic for 14 years, and he has shown no signs of ischemia and no delay of growth on his lower limbs. Thus, we could wait until his growth stopped to perform the second surgery. To our knowledge, the previous longest followup of congenital AAA after surgical repair is 48 months in a case report.^6)^ Our patient ultimately developed decreased lower ankle-brachial indexes in both limbs and felt slight pain in both limbs during exercise. At this time, we decided to perform the second operation to ensure increased blood supply to both lower limbs. Finally, the extra-anatomical bypass was successfully performed. To our knowledge, this is the first report of a second surgical repair for congenital AAA following a long period of followup.

## Conclusion

Congenital AAA is very rare, and there remains no universal treatment strategy for this condition. Longterm followup after surgery for this condition has not been previously reported. Our operative strategy of performing primary graft replacement and then performing an extra-anatomical bypass 14 years after the first operation may be one of the best treatments for congenital AAA.
